# PREDICTING ADVERSE OUTCOMES IN ED PATIENTS USING THE CLINICAL FRAILTY SCALE AND A FRAILTY INDEX

**DOI:** 10.1093/geroni/igz038.2875

**Published:** 2019-11-08

**Authors:** Kenneth Rockwood, Mohammad Pulok, Alex van der Valk, Olga Theou

**Affiliations:** 1 Dalhousie University, Halifax, Nova Scotia, Canada; 2 Nova Scotia Health Authority, Halifax, Nova Scotia, Canada

## Abstract

Our aim was to use the Comprehensive Geriatric Assessment (CGA) database to investigate whether the Clinical Frailty Scale (CFS) measuring the baseline state, and a Frailty Index (FI) based on a CGA (current state, with acute illness) can predict adverse outcomes in acutely ill Emergency Department (ED) patients. It contains CFS and FI scores on 1028 ED patients referred to internal medicine at the Halifax Infirmary between 2009-2019 (Mage 80.69 ± SD 8.28, range 57-103; 54.9% female). The mean scores were 0.44±0.14 (FI) and 5.58±1.66 (CFS). Most patients (72%) arrived via ambulance. The average length of stay was 27.0±20.5 hours. Overall, 22% were discharged home, and 63.5% had died by December 2017 with a mean survival time of 1.98±2.01 years. Controlling for age, sex, and Canadian Triage Acuity Score, the odds ratio (95% Confidence Interval) of being discharged home and the hazard ratio (95% Confidence Interval) for mortality was 0.94 (0.92-0.95) and 1.02 (1.02-1.03), respectively per 0.01-point increase in FI. For the CFS, using score ≤4 as the reference, the odds ratio and the hazard ratio were 0.70 (0.42-1.16) and 2.02 (1.51-2.69), respectively for the CFS 5 group, 0.47 (0.27-0.81) and 2.72 (2.05-3.61), respectively for the CFS 6 group, and 0.38 (0.21-0.70) and 4.67 (3.51-6.20), respectively for the CFS 7-9 group. Even controlling for acuity, both the CFS and the FI independently predict adverse outcomes in ED patients. These add prognostic information to the routinely collected ED assessments, and establish targets for care plan based on recovery to baseline.

